# From Shell Midden to Midden-Mound: The Geoarchaeology of Mound Key, an Anthropogenic Island in Southwest Florida, USA

**DOI:** 10.1371/journal.pone.0154611

**Published:** 2016-04-28

**Authors:** Victor D. Thompson, William H. Marquardt, Alexander Cherkinsky, Amanda D. Roberts Thompson, Karen J. Walker, Lee A. Newsom, Michael Savarese

**Affiliations:** 1 Center for Archaeological Sciences and Department of Anthropology, University of Georgia, Athens, Georgia, United States of America; 2 Florida Museum of Natural History, University of Florida, Gainesville, Florida, United States of America; 3 Center for Applied Isotope Studies, University of Georgia, Athens, Georgia, United States of America; 4 Laboratory of Archaeology, University of Georgia, Athens, Georgia, United States of America; 5 Department of Anthropology, Pennsylvania State University, State College, Pennsylvania, United States of America; 6 Department of Marine and Ecological Science, Florida Gulf Coast University, Ft. Myers, Florida, United States of America; ICREA at the Universitat Autònoma de Barcelona, SPAIN

## Abstract

Mound Key was once the capital of the Calusa Kingdom, a large Pre-Hispanic polity that controlled much of southern Florida. Mound Key, like other archaeological sites along the southwest Gulf Coast, is a large expanse of shell and other anthropogenic sediments. The challenges that these sites pose are largely due to the size and areal extent of the deposits, some of which begin up to a meter below and exceed nine meters above modern sea levels. Additionally, the complex depositional sequences at these sites present difficulties in determining their chronology. Here, we examine the development of Mound Key as an anthropogenic island through systematic coring of the deposits, excavations, and intensive radiocarbon dating. The resulting data, which include the reversals of radiocarbon dates from cores and dates from mound-top features, lend insight into the temporality of site formation. We use these insights to discuss the nature and scale of human activities that worked to form this large island in the context of its dynamic, environmental setting. We present the case that deposits within Mound Key’s central area accumulated through complex processes that represent a diversity of human action including midden accumulation and the redeposition of older sediments as mound fill.

## Introduction

Shell middens and mounds are complex landscape features that present a number of problems for archaeologists who wish to understand their formation. The temporality of shell features can be critical to understanding site function as well as the broader nature of how past inhabitants not only exploited their environments but also shaped them. Recent methodological advances have armed archaeologists with an arsenal of tools and techniques to more efficiently and perhaps better understand chronology of sediment formation in such features. Yet, large shell-dense sites continue to prove difficult to evaluate and interpret. Here we present some of our research at Mound Key, a large shell mound site complex in southwestern Florida. Using a combination of field and laboratory methods including coring, excavations, archaeobotany, and a large-scale radiocarbon dating program, we argue that inhabitants “redeposited” midden to construct major portions of some of the large mounds. We also argue that inhabitants reshaped their environment by reconfiguring midden mounds during the settlement’s occupation, essentially forming an anthropogenic island.

**Fig 1:**
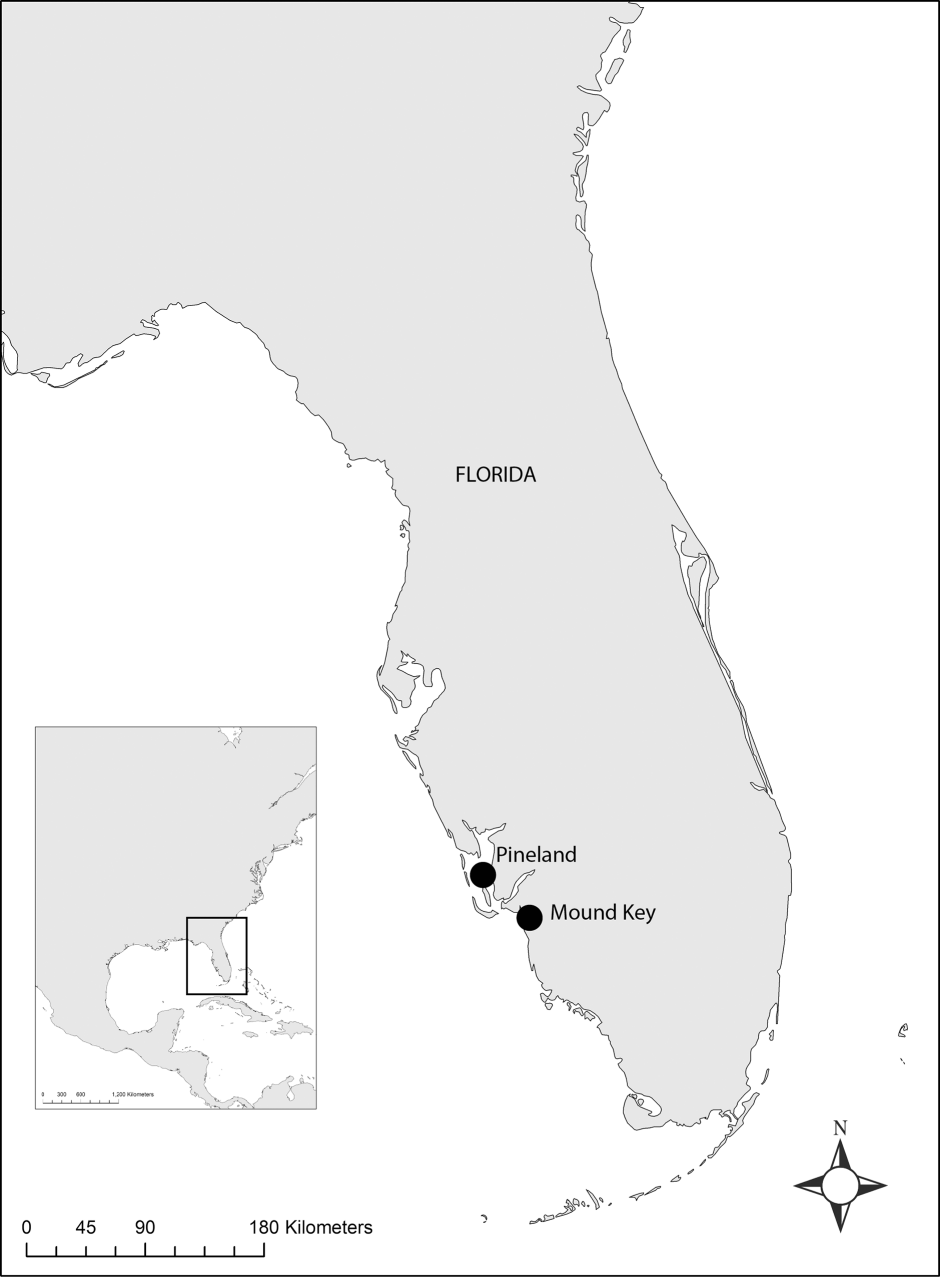
Location of Mound Key and Pineland site complexes in Southwest Florida, USA. Base map image downloaded from http://www.naturalearthdata.com/.

Mound Key (8LL2) is a 51-hectare island in Estero Bay, near Ft. Myers, Florida (Fig 1). The site was the capital of the Calusa Kingdom, a large Native American polity that controlled much of southern Florida at the time of European contact in the sixteenth century [1, 2]. From the capital, the Calusa extracted tribute from towns throughout southern Florida [1, 3, 4]. One of the more interesting facets of the Calusa is that a mainly fisher-gatherer-hunter economy supported their high degree of political complexity, with the extraction of aquatic resources being the primary subsistence activity [5, 6]. The Calusa encouraged or semi-cultivated only a few plants including chili peppers, papaya, and squash, likely tending them in small home gardens [3, 7]. Unlike other contemporary groups in the southeastern United States that were similarly organized, the Calusa did not practice maize agriculture nor staple root-crop production such as in the Caribbean islands [8, 9].

Because the extraction of aquatic resources was central to the Calusa economy, sites in the region tend to have large multiple midden mounds with mollusk shells of varying species being the most visually conspicuous component of these mounds. The Pineland Site Complex, located on Pine Island (Fig 1), is another example of such sites. It is the most extensively studied of the Calusa sites and research there demonstrates the complex interlinked history of the site’s occupation and corresponding broad-scale environmental changes [10]. Pineland may have been an earlier capital or administrative center [3, 11]. One of the key research questions of our current work is how Mound Key emerged as a capital amid other large centers, such as Pineland. To accomplish this, we first need to understand formation processes at Mound Key.

**Fig 2:**
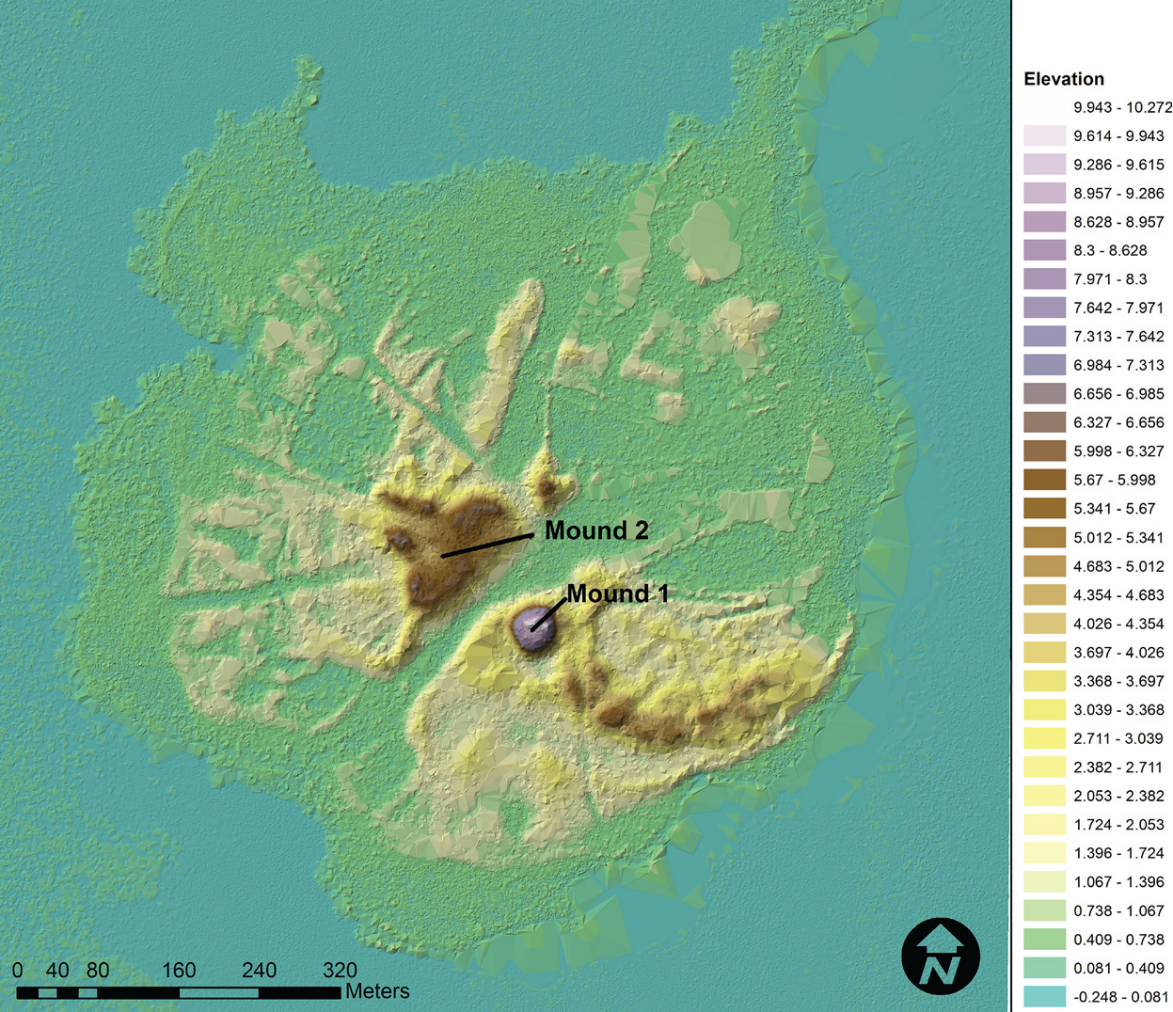
LiDAR digital elevation model of Mound Key showing the site’s prominent features. The LiDAR data were processed in ArcMap 10.2 with the LAS toolset in the program using the NAD 1983 and NAV 1988 horizontal and vertical datum respectively. All LiDAR used in the current analysis is publically available via the National Oceanic Atmospheric Association website (http://coast.noaa.gov/digitalcoast/) [15] (LAS points 202,364 over 638,841 square meters).

Although previous mapping of Mound Key provided a first look at its topographic complexity [12], newly available LiDAR (Light Detection and Ranging) data now provide the detail needed to better understand the large scale, as well as more subtle features (Fig 2). The two largest features are Mound 1 (ca. 10 m) and Mound 2 (ca. 6 m), although “mound” might not be the best descriptor if, as we contend, the Mound Key landscape was artificially sculpted, making it difficult to tell when one mound stops and another begins. For clarity and historical continuity in the archaeological literature, we retain these designations. The main canal that bisects the island separates the two mound complexes. This layout is shared by a number of sites in the region, including Pineland and Big Mound Key [10, 11, 13]. From the main canal there are several smaller canals revealed in the LiDAR. At the southernmost point of the canal are two shell-walled impoundments on each side, colloquially referred to as “water courts” by archaeologists [14]. Radiating from this core area of Mound Key are numerous terraces, ridges, elevated level grounds, and mounds.

Large shell sites, such as Mound Key and others that characterize the coasts of the southeastern United States, engender considerable debate among archaeologists regarding their formation [16–24]. As this is outlined in a number of publications, we refer the reader to Thompson and Worth [25] for a summary. Briefly the discussion centers on whether large shell deposits represent simple middens, monuments, and/or the remains of large-scale feasts. More recent scholarship is reaching consensus that simple dichotomous perspectives obscure the great variation of activities that work to create large shell sites. Attempts at midden typologies also obscure variation across different deposits [15, 23]. Despite this emerging consensus there are still obstacles, particularly in terms of exactly how we go about disentangling the complex formation processes of these sites, with recent studies employing stable isotope analysis and Bayesian modeling of radiocarbon dates [21, 22, 26].

Stein et al.’s [27] approach to assessing accumulation rates for shell sites offers one possible pathway to understanding the nature of sites in southwestern Florida. This method entails dating a large number of samples from a stratigraphic profile representing the entire depth of deposits. While this technique is, of course, an effective way to approach the problem of site formation processes, for our work it is cost prohibitive to excavate through 10 m of deposits. There have been rare occasions in southern Florida where archaeologists use this method. Recently, Luer [28] and Loger [29] use a number of radiocarbon dates to address formation processes at another Calusa site in the region, Big Mound Key (8CH10, not to be confused with Mound Key, which is 8LL2). Unfortunately, Luer and Loger average dates from levels, which leads them to interpret the nature of most deposits at Big Mound Key as the results of midden accumulation [28, 29]. Averaging dates in this manner fails to take into account prior information from the archaeological context of the dates, leading to possible misinterpretations of probability distributions of dates. This assumes that layers, as well as dated samples, represent events that should be combined, when this might not necessarily be the case. Our approach to this problem is a tiered system of confidence and evaluation of dates based first on their context, and then on type of sample (e.g., carbonized wood, species of shell).

### Recent Research

In 2013 we initiated a research program at Mound Key to understand its formation in order to shed light on the social and political history of the Calusa capital. This paper addresses several basic questions related to the site’s formation from a geoarchaeological perspective. Specifically, our research focused on three central questions:

1. What formation processes best characterize the large midden-mound deposits at the site?
2. What is the overall chronology of deposition at the site (i.e., occupational history)?
3. What is the variation in timing and depositional processes across the site?

While there is some overlap among these three research questions, each of them addresses a fundamental aspect of the creation of the Mound Key landscape in terms of its dynamics, structure, and timing—information on these three dimensions forms the core of geoarchaeological research [30]. At the end of this paper, we contextualize our geoarchaeological findings with what we know historically and archaeologically regarding Mound Key, Pineland, and the Calusa in general.

## Methods

We employed three different methods to gather data that could be used to evaluate the structure, dynamics, and chronology of Mound Key. These methods included coring, test and block excavations, and an intensive radiocarbon dating program. All permits were granted by the Florida Bureau of Archaeological Research (permit #1213.031) and the Florida Department of Environmental Protection. The individual in this manuscript has given written informed consent (as outlined in PLOS consent form) to publish these case details.

### Coring

To extract sediment cores we used a JMC Environmentalist’s Sub-Soil probe. This is a manual slide hammer operation corer. Sampling with this instrument follows a basic process of inserting a clear plastic liner into the sampling tube, placing the tube over the sampling area, and then sliding the hammer into the ground until it reaches its maximum depth. Extraction of the sampling tube can be accomplished in one of two ways, either by a pedal jack or simply by removing the tube by hand. We used hand removal most often, except in wet areas where the suction of the tube prevented easy extraction, necessitating the use of the jack. Each sampling tube produced a core sample that was 91.5 cm in length and 3.05 cm in diameter. We used a number of extensions to increase core length to 3.66 m. We made every effort to minimize fall into the core hole once we extracted a section; however, some fall was unavoidable. This slump, however, is easily identified as the top most strata of the core sections. These materials once identified were then discarded. We trimmed core tubes flush to the sediment surface while in the field to avoid disturbance of the strata during transport.

**Fig 3:**
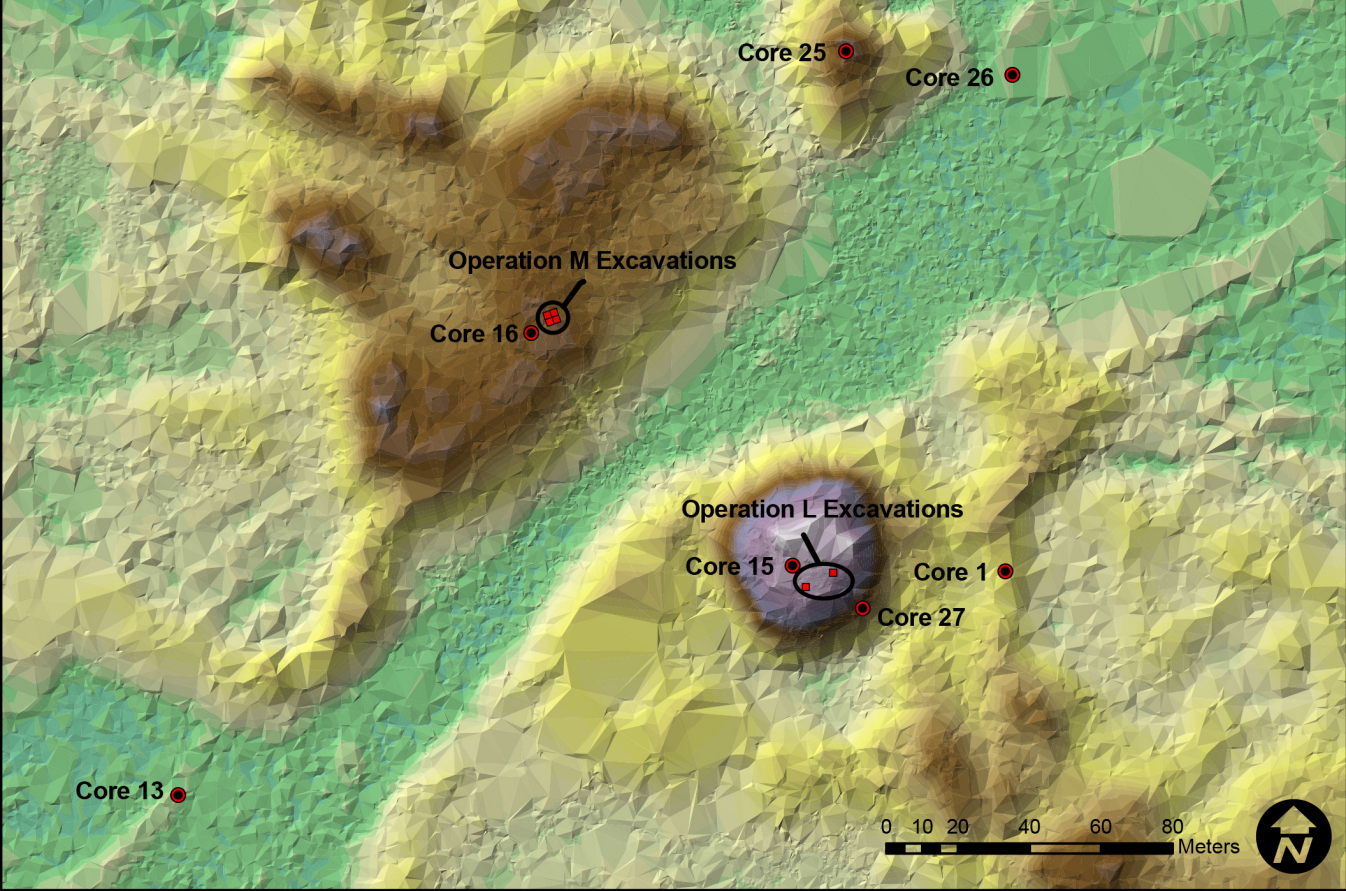
Location of cores and excavation units described in the text in the vicinity of Mound 1 and Mound 2. The elevation scale (in meters) for this image is the same as the one used in Fig 2.

In all, we extracted cores from 28 locations across the site; however, we only report herein on cores for which we have radiocarbon dates (n = 7). The locations of these cores are indicated in Fig 3. Our general strategy was to sample landforms across two transects that roughly bisect the site along two directions. We did not conduct transects in straight lines nor at fixed intervals because the site is not perfectly oriented to the cardinal directions, nor are the features evenly distributed.

We analyzed all cores at the Laboratory of Archaeology at the University of Georgia. This entailed bisecting and examining one half of the core tubes, photographing each section, and recording the stratification on a standardized form. For our initial evaluation of the cores, we recorded the color using a standard Munsell color book and noted any changes in stratification, particularly with respect to shell density. We used nested sediment geological sieves (>4mm, >2mm, and < 2mm) to determine the grain size distribution of each stratum and the percentages of shell in each layer (i.e., the combined total of each nested sieve). We then weighed and recorded the percentage of shell using a standard Geotechnical Gauge for estimation. We bagged all samples and will curate them at the Florida Museum of Natural History for future reference.

### Excavations

We conducted excavations in 2013 and 2014 with the purpose of identifying structural remains associated with both the Calusa and Spanish occupations. We quickly realized, however, that the location and dating of these features and surrounding midden materials provide a complementary dataset to our cores by which to evaluate the nature of site formation processes. Our excavations focused on the tops of the two largest mounds (1 and 2). On Mound 1 we excavated two 2m-x-2m units. On Mound 2 we excavated four 2m-x-2m units in a contiguous block (see Fig 3). Both of these excavations were shallow and had a maximum depth of 60cm below the surface. The research team, using a level-locus system to avoid mixing strata within arbitrary levels, excavated all units in 10-cm levels by trowel and pedestaled and removed features one level at a time. We also draw on data from excavations conducted previously by Torrence et al. [12] that are unreported in a formal publication or report. These older excavations were small 1m-x-1m test units also excavated in 10-cm levels using a level-locus system. The radiocarbon results from the excavations here provide a supplementary dataset to the cores. Therefore, we do not offer a full description of the excavation results in this paper, as this is the subject of another forthcoming publication.

### Radiocarbon Dating

From the 2013 and 2014 excavation units and cores, we ran a total of 53 radiocarbon dates. In addition, in our analysis we incorporate nine dates that were analyzed previously by Beta Analytic, Inc., on samples from the 1994 work. Our sampling strategy for the dating includes multiple samples from the cores, samples from features, and samples from general midden context from the excavations. Dated sample materials included shell, deer bone, sediments, and carbonized wood from post features. The Pennsylvania State University’s Environmental Archaeology Laboratory identified all carbonized wood prior to submission, with the results ultimately guiding sample selection for dating. University of Georgia’s Zooarchaeology Laboratory identified the bone selected for dating.

All of the Accelerator Mass Spectrometry (AMS) radiocarbon measurements were carried out at the Center for Applied Isotope Studies (CAIS) facility at the University of Georgia. For these analyses, the procedures followed the standards outlined by Cherkinsky et al. [31] that are paraphrased here in summary form. We leached shell samples with diluted HCl in an ultrasound bath to remove surface contamination. Samples were then washed and dried in a vacuum with 100% phosphoric acid to recover carbon dioxide. For carbonized wood, we treated samples with 1N HCl at a temperature of 80°C for one hour and then washed the samples with deionized water on the fiberglass filter and rinsed with diluted NaOH to remove possible contamination by humic acids. We then treated samples with diluted HCl again, washed with deionized water and dried at 60°C. For our bone samples, we cleaned them by wire brush and washed them in an ultrasonic bath. After cleaning, we gently crushed the dried bone to small fragments. We then treated the sample with 1N HCl at 4°C for 24 hours and then filtered the residue through a fiberglass filter. We then successively rinsed the sample with deionized water, 0.1N NaOH, deionized water and diluted HCl. Then under slightly acid conditions (pH = 3), we heated the precipitate at 80°C for 12 hours to dissolve collagen and leave humic substances. We then filtered the collagen solution to isolate pure collagen and freeze dried it and then combusted at 575°C in an evacuated/sealed Pyrex ampoule in the presence of CuO.

Following Cherkinsky et al. [32], the samples were cryogenically purified from other reaction products and catalytically converted to graphite. The CAIS laboratory used the CAIS 0.5MeV Peletron accelerator to measure graphite ^14^C/^13^C ratios. The laboratory then compared all the sample ratios to the ratio measured from Oxalic Acid I standard (NIST SRM 4990). For our work, the ratio of ^14^C/^13^C ratio is the measured parameter on the accelerator, which is normally used with reference to the same ratio of a primary standard with the subtraction from this ratio of the blank measurement. We present all the quoted uncalibrated dates in radiocarbon years before 1950, using the ^14^C half-life 5,568 years.

After initial evaluation of our radiocarbon dates, we then performed a Bayesian analysis of the dataset to avoid overestimations of time, as well as earlier estimated starting dates for the onset of site occupation (see discussion in [33]). To construct our models, we use prior information such as the location of features (i.e., *a priori* beliefs) regarding the nature of the dates from Mound Key (see discussion in [33]). The basic tenets of Bayesian modeling and Bayes’ Theorem regarding its use for constructing radiocarbon chronologies are outlined in numerous publications [26, 34–39].

Our analysis incorporates two simple phase models in which probability distributions are grouped as unordered events, one for dates associated with features (i.e., post molds) on the tops of the mounds and a second one for the site as a whole, using all dates. In each of these models radiocarbon dates are grouped together both as a series of ordered events (if prior information is available) or as a series of unordered events, both with a boundary start and end. To calculate the duration of mound tops (Mounds 1 and 2) as well as overall site occupation, the span function was used in OxCal. Solutions for each model were evaluated based on convergence (i.e., values greater than 95), as well as agreement for individual dates (A), the model, (A_model_), and the overall agreement, (A_overall_), with values over 60.0 indicating significant agreement (see discussion in [40]).

## Results

S1 Table contains all the corrected and calibrated dates for Mound Key listed by lab and sample identification number. In this section, we present a brief description of the analysis of cores and excavations; however, our primary purpose is to present information regarding the context of our radiocarbon dating dates, as well as the results of our dating program at the site.

**Fig 4:**
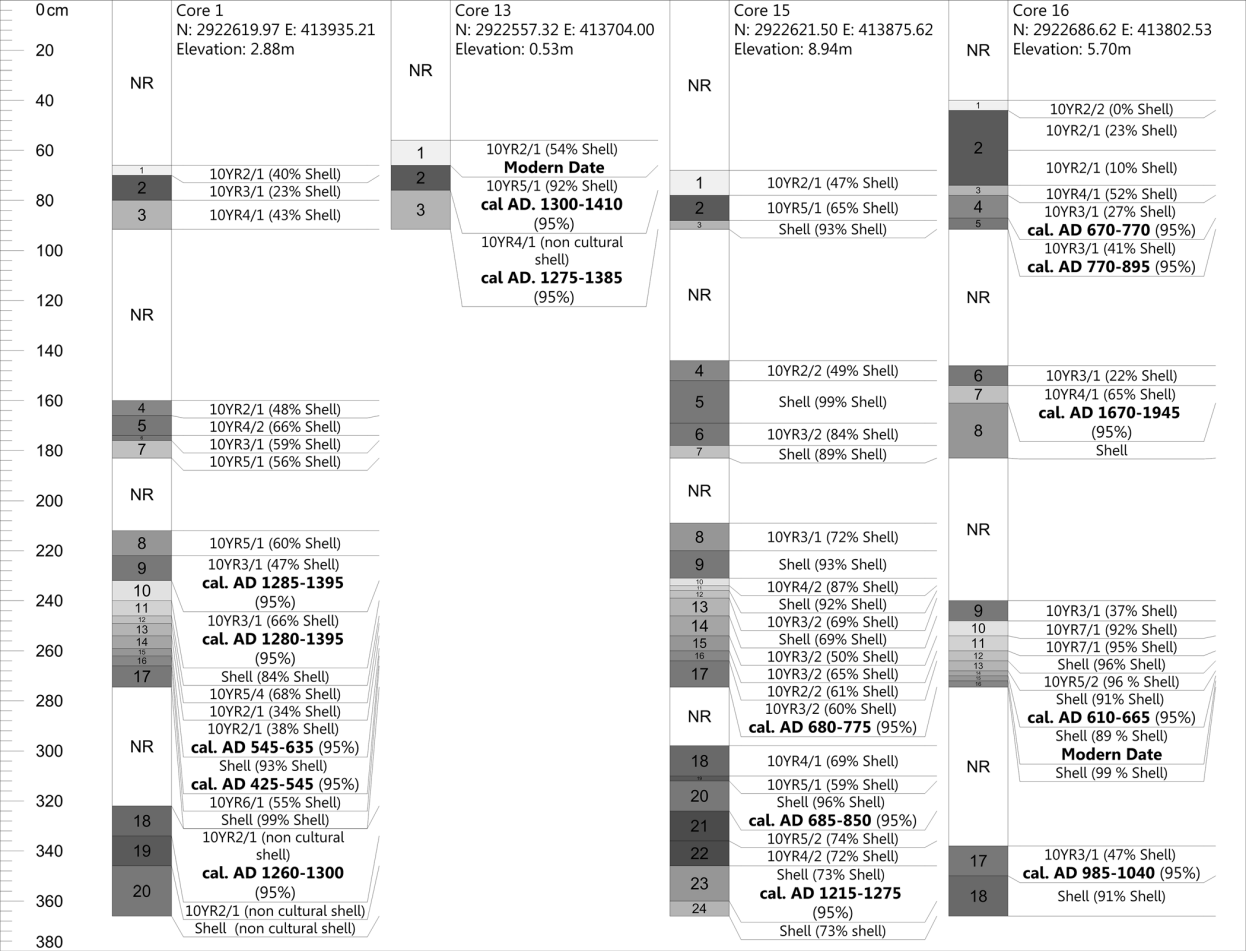
Cores 1, 13, 15, and 16 and their associated AMS dates from Mound Key. Cores are labeled in stratigraphic order from surface to bottom sequentially with NR meaning no recovery due to compaction. Only shell percentages are shown in the figure (e.g., if 40% shell is shown in figure the remaining 60% would represent other). All dates are reported as their modeled values.

**Fig 5:**
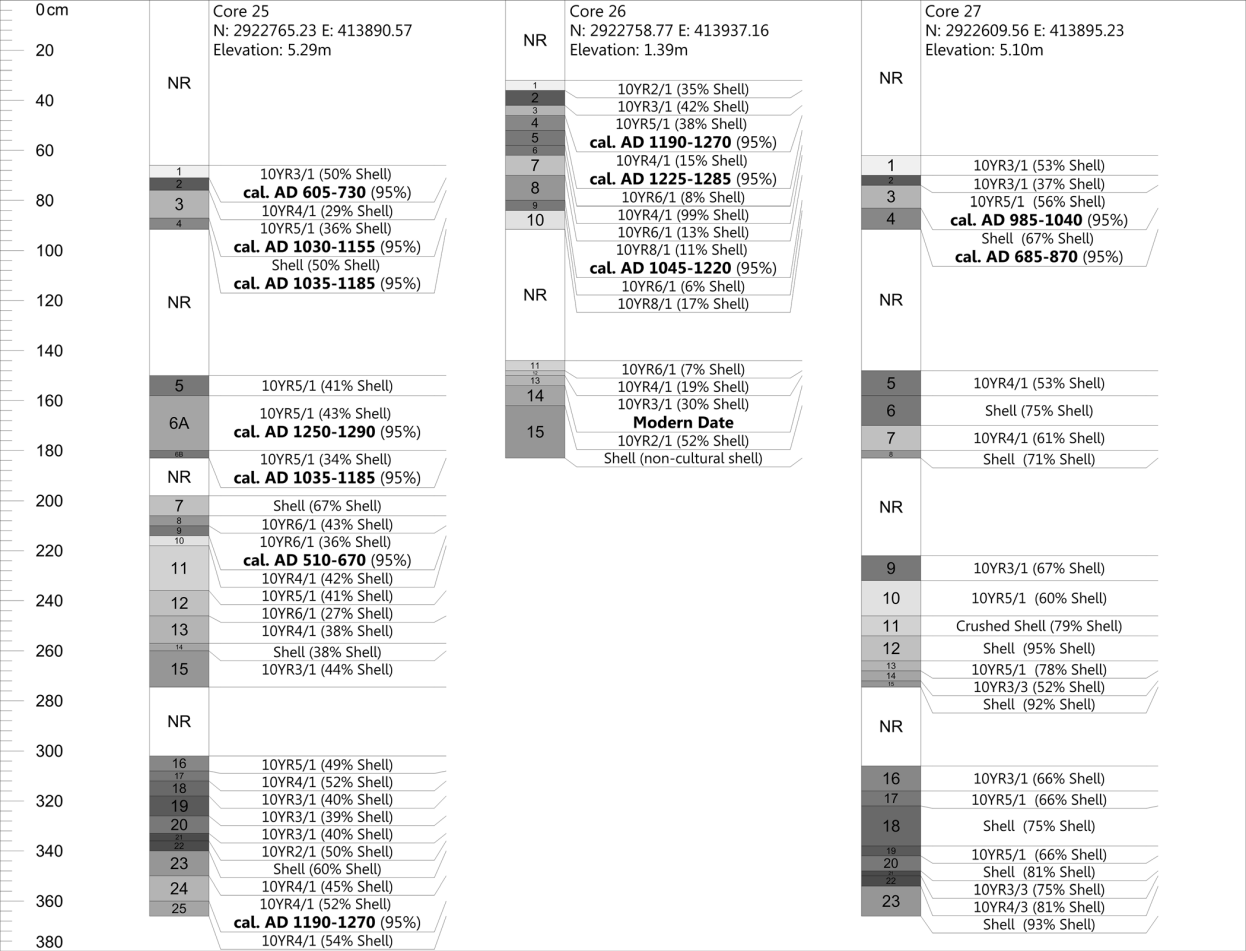
Cores 25, 26, and 27 and their associated AMS dates from Mound Key. Cores are labeled in stratigraphic order from surface to bottom sequentially with NR meaning no recovery due to compaction. Only shell percentages are shown in the figure (e.g., if 40% shell is shown in figure the remaining 60% would represent other). All dates are reported as their modeled values.

We do not provide full descriptions here of all of the cores or of the excavations, but instead offer details regarding these as they relate to our radiocarbon dating program and the specific research questions outlined in this publication. In total, we ran 30 AMS dates from seven different cores (Cores 1, 13, 15, 16, 25, 26, 27) (Figs 4 and 5).

### Core Chronologies

Core 1 (see Fig 4) is from a 3.09-m high shell ridge adjacent to Mound 1 (see Fig 3). This core has varying strata of dense shell deposits interbedded with layers containing more detrital sediments and bone (e.g., bone, sand, and silts). In the lowest levels of this core are non-cultural sediments characteristic of either sub-tidal or tidal environment deposits, similar to those found in the bay today. On top of these are organic peat-rich layers that formed once this area was isolated from the bay by other shell midden-mound deposits that blocked the open bay waters from this area. Several dates from this core indicate disordering of the sediments. The bottom-most date is *cal*. *AD 1260–1300* with two dates in the cal. AD 400 to 600 range above it, followed by another two dates in the cal. AD 1200–1400 range. We took this core adjacent to the 1994 excavation unit, C-1, which had dated samples from three levels correlating with strata above the dated ones in the cores. These C-1 dates are also out of sequence and are in the cal. AD 400 to 800 ranges (see S1 Table).

Core 13 (see Fig 4) is from the western shell enclosure at the southern end of the site (see Fig 3). This enclosure and its eastern counterpart (see Fig 2) are hypothesized to be storage areas for surpluses of live, aquatic animal foods; however, no empirical research has yet substantively tested this idea. This core exhibits only three strata; a top organic peat-rich layer that returned an expected modern date, a second stratum of shell with a top and bottom date *cal*. *AD 1300–1410* and *cal*. *AD 1275–1385* respectively (see Fig 4), and a lowest stratum that is similar to the bottom deposits of Core 1, which is characteristic of either sub-tidal or tidal deposits found in the bay today.

Core 15 (see Fig 4) is from Mound 1 (see Fig 3) and represents only the top 3.7 m of the mound’s total ca. 10 m of sediments. It exhibits generally alternating strata of dense shell and layers in which non-shell sediments show greater contributions. The dates from the core again exhibit reversal with a date of cal. AD 1215–1275 situated below two dates in the cal. AD 600 to 800 ranges.

Core 16 (see Fig 4) is from Mound 2 (see Fig 3) and represents only the top 3.7 m of this mound’s ca. 6 m of sediments. It has similar strata to Core 15 and also demonstrates a reversal of dates; however, one modern date came from section 3 of this core. It is possible that this core was contaminated during its extraction or that the modern date is the result of significant bioturbation in the area.

Core 25 (see Fig 5) is from the ca. 5-m-high Mound 3, which is just northeast of Mound 1 (see Fig 3). This core, along with Core 1, provides the best insight into midden-mound formation, as cores were extracted easily and there was less compaction in these sections than in other cores. Similar to Mounds 1 and 2, this semi-conical mound exhibits complex alternating groups of strata of dense shell deposits in contrast to deposits containing more non-shell sediments. Just as with the other cores, this one exhibits reversal of the dated materials. The upper section (i.e., the upper 91cm) provides the best evidence that the reversal of dates is best explained by construction processes. Our dating of the top-most stratum, which is free of potential contamination by insertion and removal of the core tube sections, exhibits an older date while the strata just below date to more recent centuries.

Core 26 (see Fig 5) is from a small sand rise of 1.39 m just east of Mound 3 (see Fig 3). This core had only minimal amounts of shell. We thought at first that the alternating sand layers in the core might represent storm surge deposits; however, after retrieving a modern date from the lowest level dated, we reconsidered this explanation. We now believe that this small mound represents a spoil pile from a previous unrecorded excavation. It is perhaps from a prior visit by the antiquarian C. B. Moore, who excavated near this mound [41].

Core 27 (see Fig 5) is from the slope of Mound 1 (see Fig 3). This core had similar stratification to Core 15; however, the two dates for Core 27 appear to be in chronological order.

### Excavations and Mound-top Chronologies

**Fig 6:**
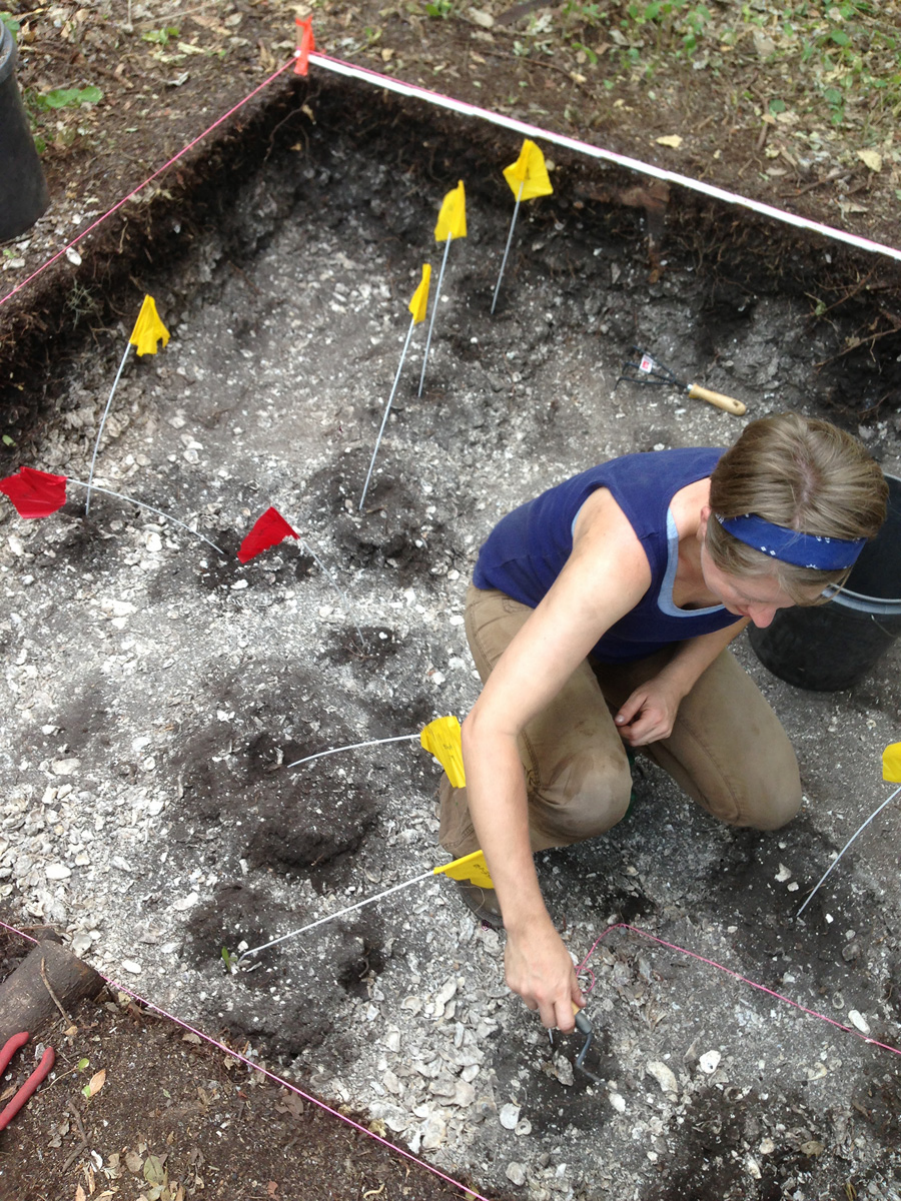
Excavation of M-1 on Mound 2 showing the clear post features in the process of excavation.

The 2013 and 2014 excavations revealed a number of structural features. In all, we identified a minimum of 45 post-mold features from the units on Mound 1 and 117 post-mold features from the units on Mound 2 (Fig 6). Shell midden materials surrounded all of the features. Throughout the excavations on Mound 2, we recovered ceramics associated with both the pre-contact Calusa occupation and ceramics related to Spanish contact. In some instances, ceramics of both European origin and Calusa manufacture were included in post fill, offering further chronological information on the position of these features in time. In contrast, the Mound 1 excavations produced no in situ definitive artifacts of European manufacture.

Our recovery of post-mold fills and the surrounding midden material provided us with the opportunity to date not only carbonized wood from the fill, but also the surrounding matrix (see S1 Table). The excavations show that the very topmost deposits associated with the post molds that are visible in the profiles on Mound 1 are missing. It is possible that either these layers were eroded and or, as we discuss below, obliterated by subsequent constructions on the tops of the mounds.

Wood identifications indicated that pine (*Pinus* sp.) was the most ubiquitous taxon recovered in the post-mold sediments. Therefore, we chose carbonized wood of this taxon for our radiocarbon analysis. We deem that the “old wood” problem [42] is highly unlikely to present an issue for radiocarbon dating in this climate and landscape. Mound Key is situated in Florida’s subtropical moist forest bioclimate zone [43], which is characterized by relatively mild winters and generally high temperatures and rainfall (annual rainfall exceeding 1500 mm). Rates of natural decay of organics are high under these conditions, and the abundance of biological agents of wood decay (e.g., termites and fungi) contributes to the rapid pace of decay. These conditions thus are not conducive to long-term survival of fallen or standing deadwood, very much unlike the situation in the arid southwest where the “old wood” problem was first described. For the same reason, we consider it unlikely in this case that old salvaged wood will have survived enough intact for reuse in building construction (the dated charcoal derived from post features) at least not for extended periods of time.

In order to date the surrounding midden we dated two white-tailed deer (*Odocoileus virginianus*) bones, as well as clam shells, *Mercenaria campechiensis* (southern quahog). Previous studies favor this species for dating over others such as oyster (*Crassostrea virginica*) or Florida fighting conch (*Strombus alatus*), because these latter two produce unreliable dates [31, 44, 45].

**Fig 7:**
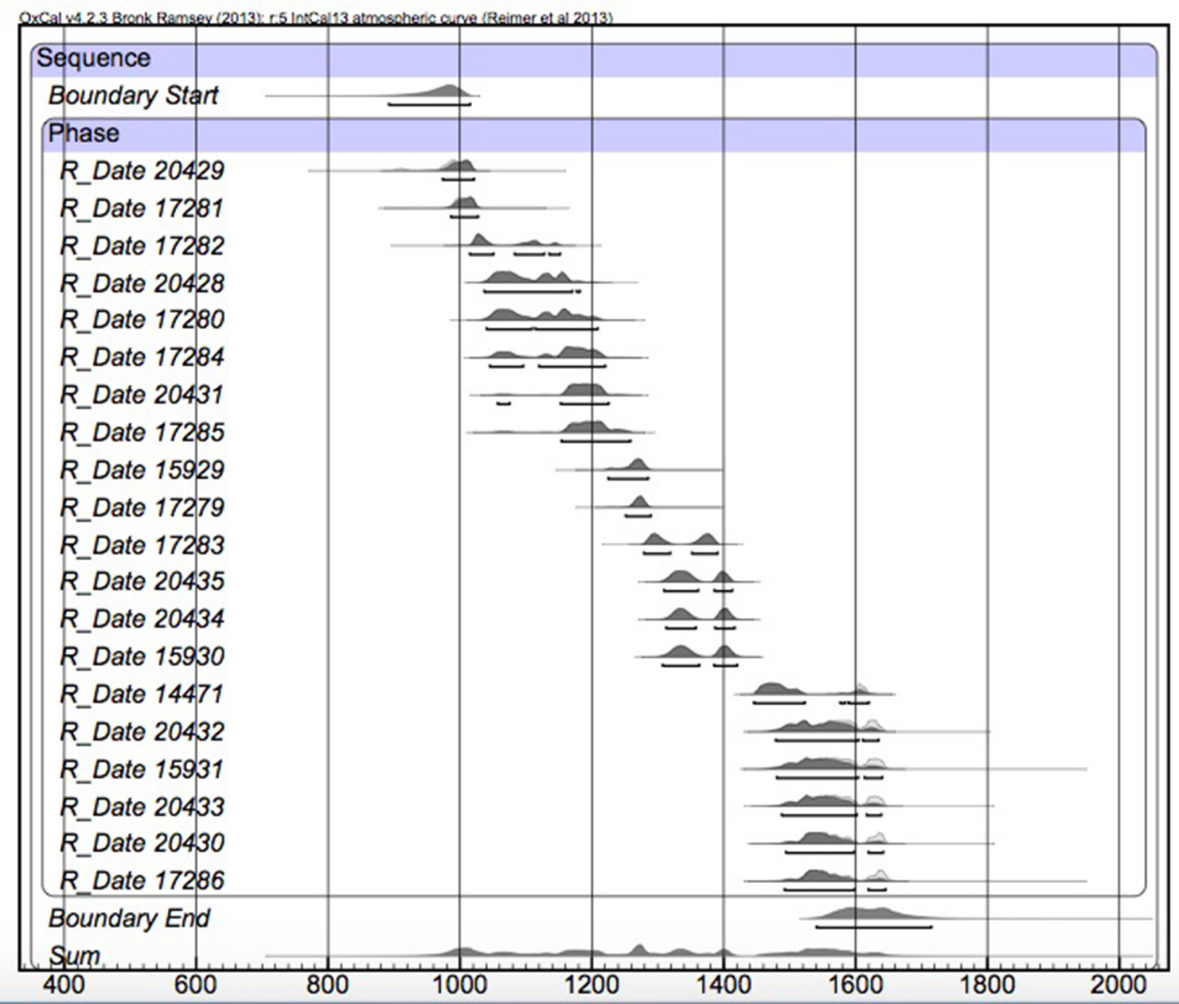
Probability distributions of Bayesian modeled dates from post molds from the tops of Mound 1 and Mound 2. All corresponding provenience information for each date can be found in S1 Table.

The features excavated from roughly the upper 60 cm (the limits of the excavations) of the tops of Mound 1 and Mound 2 represent the most securely dated contexts at the site. They also provide an opportunity to examine the nature of mound-top activities at the center of the site. We dated 20 features (19 post-molds and one “fallen” post feature) via AMS from both mounds, which we include in this analysis (Fig 7). The Bayesian model shows good agreement between the radiocarbon dates and phase modeling of activities associated with the tops of both mounds (A_model_ = 99.1; A_overall_ = 99.1). For the tops of both mounds, the model estimates an activity start date of *950–1005 cal*. *AD* (*68% probability*) and *890–1015 cal*. *AD* (*95% probability*) and an activity end date of *1570–1660 cal*. *AD* (*68% probability*) and *1540–1715 cal*. *AD* (*95% probability*). The span of activities for the tops of both mounds is *585–695 years* (*68% probability*) and *545–785 years (95% probability)*.

### Overall Site Chronology

**Fig 8:**
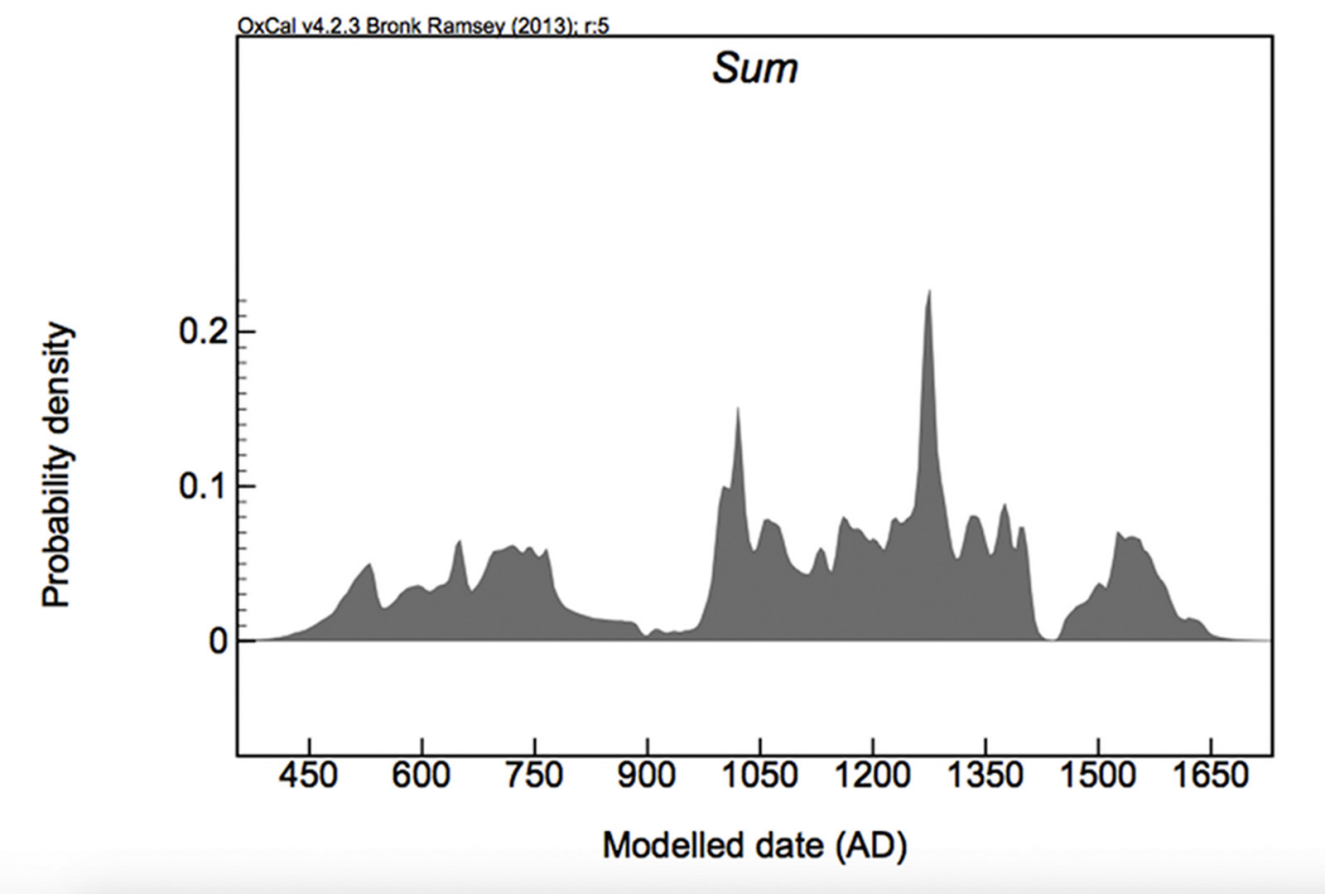
Summed probability of all modeled dates from Mound Key showing the possible gap in occupation. All corresponding provenience information for each date can be found in S1 Table.

If we examine the site as a whole, using all available dates, again our Bayesian model shows good agreement (A_model_ = 89.3; A_overall_ = 81.2) with only one date showing poor agreement with the model. This date happens to be the earliest at the site with a reported date of cal. AD 285–550 at the 95% probability (Beta-245276). The model estimates an activity start date of the occupation at the site as *460–530 cal*. *AD* (*68% probability*) and *400–540 cal*. *AD* (*95% probability*) and an occupation end date of *1550–1625 cal*. *AD* (*68% probability*) and *1530–1670 cal*. *AD* (*95% probability*). The span of activities for the site as a whole is *1045–1155 years* (*68% probability*) and *1015–1230 years (95% probability)*. When we examine the sum probability of these dates for the site as a whole, there is a gap in the record *ca*. *800–950 cal*. *AD* for the modeled dates (Fig 8).

## Discussion

### Complex Issues in Dating Coastal Sites

There are many complex issues when dating island and coastal sites. Marine reservoir corrections, old wood problems, the reliability of certain shell species, and environmental uptake of older carbon should all be considered in any dating program. In addition to these concerns, the importance of the context of what is to be dated cannot be overstated, as well as its relationship to other artifacts and stratigraphic documentation. One rationale for selecting shells is that they do not move as much as other types of smaller samples in post-depositional environments; these are thought to represent secure samples to date. If problematic shell species are avoided, then the dating of general-midden shells can be a valid and successful dating strategy when combined with the dating of features, especially in the context of long-term research projects that conduct extensive hand excavations and detailed plan-view and stratigraphic documentation, such as is the case at Pineland [10]. However, as we point out, much of the basic science for specific species and contamination effects for certain regions is poorly understood [31, 44, 45]. In addition to these issues is the possibility that occupants rearranged large portions of the deposits over time. Such activities would also apply to carbonized wood and bone from such contexts. This is exactly what we argue accounts for the reversals of dates in the cores, as well as many of the older general-“midden” context dates surrounding younger posts.

Our dating program at Mound Key focused on three key contexts that included materials from the cores, materials from general-midden contexts, and materials from feature context. Obviously, in some instances at sites with large-scale shell deposits and few or no known features, researchers are left with few choices in what materials they can submit for dating. This was the case for our samples from the cores where we had few options. In the end we dated marine shells (*M*. *campechiensis*), as well as small fragments of carbonized wood. We note that in dating samples from cores there is always the possibility that slump from the previous section may contaminate the next core section; this may be what happened with one of our cores (Core 16). However, given the fact that we have reversed dates from the first section of one of the cores (Core 25), where such a situation could not occur, as well as reversed dates from excavation contexts, we argue that contamination of this kind does not account for the overall pattern observed. In addition, researchers working at other sites in Florida who use the same methods of dating core materials have not experienced the widespread reversal of dates that we have at Mound Key [26, 46]. Thus, while productive, the dating of the cores, as well as those dates from general-midden contexts, must be interpreted cautiously.

By far the most secure dates from Mound Key thus far come from our excavation of features on the tops of the mounds. The limitation, of course, is that when sites have over five meters of deposits (over nine meters for some areas of Mound Key) it becomes a difficult, time consuming, and expensive process to excavate all the way to the base of deposits. Therefore while we are most confident in our evaluation of dates from the features, they become more meaningful when we compare them to our core and general-midden dates. These data all underscore the fact that we cannot assume that midden formation is a linear process [16, 20, 47–49], and that we must understand the complex nature of shell deposition to properly contextualize our dating programs.

### From Shell Midden to Midden-Mound

Despite the complex issues noted above regarding the dating of island and coastal shell sites, the results of our radiocarbon program at Mound Key reveal much about the nature of the dynamics, structure, and timing of site formation and occupation, which helps us to address our original research questions regarding site formation processes of the midden mounds, overall chronology, and variation in depositional processes.

In Betty Meehan’s [50] foundational book, *From Shell Bed to Shell Midden*, she documents the behavioral processes of shellfishers and how mollusks are collected from their beds, prepared for consumption, and consumed, their shells then being disposed of and creating archaeological sites. This wonderful book provides great insight into the creation of archaeological shell midden sites by Anbarra hunter-gatherers in the Northern Territory of Australia, including the rearrangement of previously deposited shell at campsites [50]. Indeed, in other areas of the world, such as the Ertebølle shell middens in Denmark, researchers are beginning to realize that the movement of shell deposits during a site’s occupation may have been greater than previously thought [51]. Building on this work, researchers have come to understand that a number of different additional processes can contribute to the shaping and reshaping of deposits, particularly once we begin to consider the ritual and political aspects of such depositional acts [52]. Our work at Mound Key suggests that these same processes might also operate on a grand scale. For the largest deposits at Mound Key (i.e., Mound 1 and Mound 2), we suggest that to a large degree (at least the top 3.7 m) these structures were formed through what we call redepositing midden. This entailed the mining and moving of old midden to form specific, preconceived structures.

**Fig 9:**
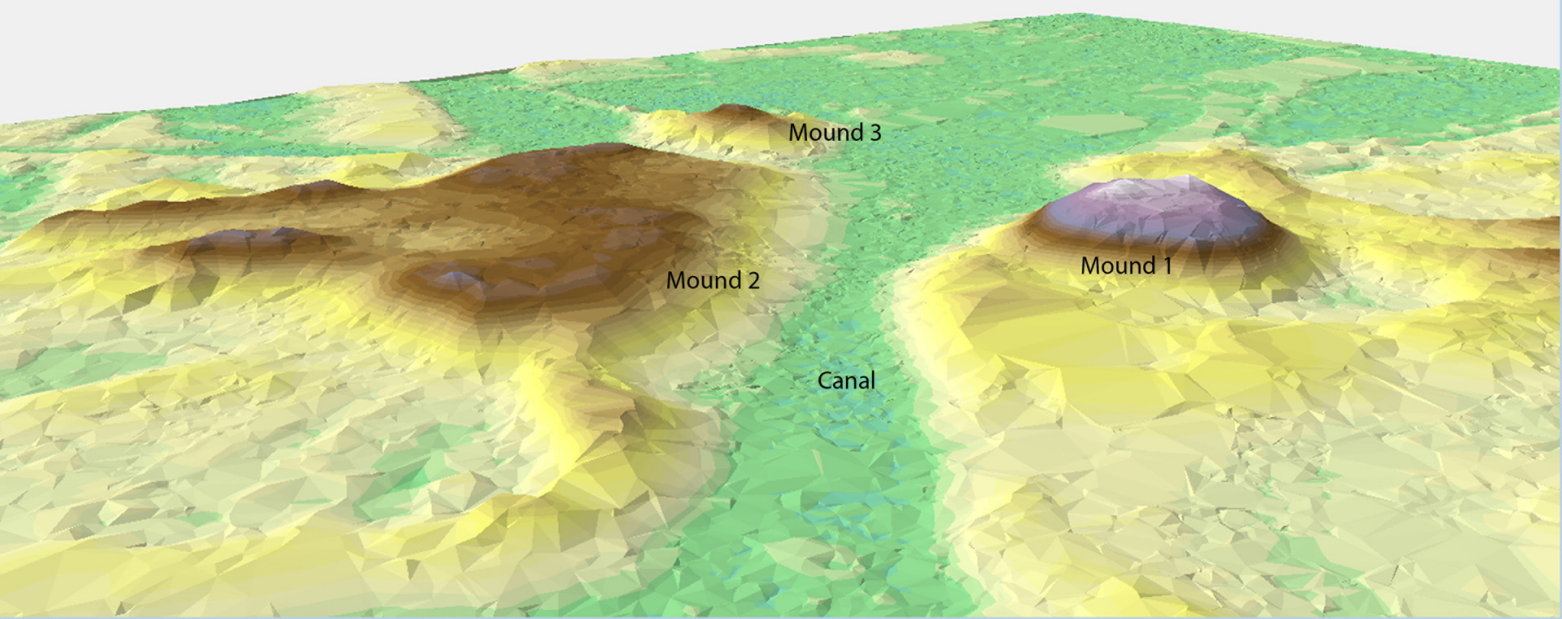
3D digital elevation model (x1.5 vertical exaggeration) of Mound Key LiDAR showing the topography of Mound 1 and its relationship to the canal and Mound 2 and Mound 3.

We base this conclusion on three lines of evidence. First, based on the modeled dates, Mound 1 and at least a *certain portion* of the top of Mound 2 reached their approximate maximum heights, ca. 10 m and ca. 6 m, respectively, sometime between cal. AD 1000 and 1200, serving as platforms for structures, judging from the distribution of post features on their summits. Second, cores from these mounds, as well as others (e.g., Mound 3), show reverse chronologies of dated materials, suggesting that there was massive rearrangement of deposits over a substantial area of the central part of the site, which includes these two mounds. Finally, the slope of Mound 1 often exceeds 40 degrees and appears as a purposefully constructed truncated cone (Fig 9) and its placement conforms to a replicated pattern identified in other sites in the region indicating some structural guidelines for the placement of such midden mounds [15, 53]. The formality of this mound seems to indicate some intentionality regarding its final form; however, we note that form can belie the complex activities that worked to create it.

While the redeposition of midden seems to best describe the formation of at least the upper 3.7 m portions of the large midden-mounds at the site, we suggest that earlier in the occupational history of Mound Key, most of the shell-midden deposits likely were the result of subsistence collection, consumption, and disposal, followed by an occupational decline. It is possible that during its earliest occupation there was little redepositing of midden, although given that the cores on top of the tallest midden-mounds (Mound 1 and 2) did not sample deposits below 3.7 m, we cannot rule out other potential chronological reversals and midden redeposition of these deposits for at least these mounds. However, we do have several cores that sample to pre-occupational surfaces from other midden-mounds, as well as dates for theses cores—although these date slightly later in time than the earliest dates for the two largest mounds. Currently, our interpretation of this pattern is based on the sum probability distribution of all the radiocarbon dates. The distribution indicates that site occupation began probably sometime between AD 300 and 500 and began to decline around the latter half of the eighth century (see Fig 8). While it is desirable to have a large data set when looking at the summed probability for a region [54], our site level data are sufficiently large enough and correlate with other archaeological data sets and climate that they provide a good starting point to discuss overall trends in the occupation of the site.

Walker has argued [55, 56] that Tanner’s [57, 58] Gulf of Mexico and Denmark sea-level records, along with Stapor et al.’s [59] more local one, are all relevant to southwest Florida. In the most detailed of these [58], the fifth century is characterized by a brief lowering of water that precedes the final peak (ca. AD 500) of the Roman Warm Period. Such a lowering could have exposed a sand bar, oyster bar, and or mangrove island, creating relatively high ground to be taken advantage of by Mound Key’s first inhabitants.

The remainder of this early occupation correlates with the regionally known Caloosahatchee IIA period (ca. AD 500–800) and the broader cooling episode known as the Vandal Minimum [55]. It is during this time period that sea level may have been overall lower relative to pre-IIA and to “modern” (i.e., 20^th^-century) levels, based on research at Pineland and other sites in the Pine Island Sound region [10]. Moreover, there may have been three successive sea-level lowerings. The Tanner [58] sea-level record shows an abrupt regression ca. AD 550 to 600, a minor transgression ca. AD 600 to 650, another significant regression ca. AD 650 to 700, then a third brief transgression, followed by a third sharp regression, reaching its lowest point at ca. AD 850 [55]. All but the last of these fluctuations, the one centered on AD 850, are evidenced in Pineland’s deposits based on multiple lines of evidence [10].

The dramatic reduction in local population and possible subsequent abandonment of Mound Key around AD 850, as suggested by the modeled radiocarbon dates (see Fig 8), coincides with the third and lowest of the sea-level drops noted above. What is most interesting about this gap in occupation is that its timing matches the inferred abandonment of Pineland. In their study of Pineland, Marquardt and Walker [10] note that there are no deposits that conclusively date between AD 800 and 900, correlating to the most significant regression in the series of sea-level regressions. Their hypothesis for this abandonment is that the regression series resulted in “a greatly diminished fishery” in the shallow inshore Pine Island Sound, prompting a move to better fishing grounds [10]. It is quite conceivable that a similar process unfolded at Mound Key, located as it is in another shallow estuarine setting.

By cal. AD 950 it appears that the Calusa once again occupied Mound Key (see Fig 8). It is during this time that sea levels once again returned to higher stages. The construction of some of the upper portions of the two highest mounds at this time indicates that the redeposition of old midden might have been a rapid event. Once constructed to approximately their current height (plus or minus ca. 0.5m), Mound 1 and at least some portion of the summit of Mound 2 were stable surfaces for the next several centuries. In addition, it is possible that today’s basic form of Mound Key with its two primary mounds separated by a canal was established at this time (see Fig 9). We further hypothesize that the deposits that were once near the main canal are the old midden material used to form the tops of Mounds 1 and 2. There is tentative evidence that the rest of the site grew in an accretional manner out from this core area; however, at this point our only indication of this are dates from Core 13, located on one of the shell enclosures that returned ages that post-date the initial occupation of the summits of Mounds 1 and 2 (see S1 Table).

Again, the pattern of reoccupation of Mound Key mirrors the situation at Pineland. As with the similar timing of abandonment, the Calusa reoccupied Mound Key and Pineland at essentially the same time (ca. AD 900–1000). Further, returning Pinelanders altered their habitation pattern from one that previously paralleled the shoreline to one oriented perpendicular to it and parallel to a central canal. Low lying areas were managed for water control while mound-top habitation intensified. And, although there is no absolute chronology for the Pine Island Canal (which begins at Pineland), the Calusa likely constructed much of it in addition to other water features at this time as well due to the rising sea and ground-water levels [11].

Placing what we know now about Mound Key and its similarity to Pineland in the broader regional context reveals interesting dynamics regarding coupled socio-ecological entanglements. The AD 800–900 gap in the record is also recorded at two other sites in the region, Josslyn Island and Galt Island [11]. In contrast, Useppa Island has extensive ninth and tenth century deposits, suggesting that groups moved to islands situated near deeper waters during this time of lower sea level [10, 11]. Given the coincident occupational histories of several Calusa sites in the region, it appears that the shallow waters of the sounds and bays of this area and their resources were susceptible to broad scale, erratic lowering in sea level that accompanied the Vandal Minimum cooling episode. However, once sea levels returned at the onset of the Medieval Warm Period, groups returned to sites such as Pineland and Mound Key.

While in general the Medieval Warm Period accompanied the onset of greater productivity in the estuaries of the region [10, 55], the Calusa did not merely reoccupy their previous homes. The picture that is emerging from Mound Key and Pineland is that once groups returned to these sites they transformed the landscapes on a grand scale. At least for some of the upper portions of the two largest midden-mounds at Mound Key, it appears that there were large-scale labor projects involved in the redeposition of midden deposits for mound construction. Given the dates for abandonment and subsequent occupation of mound-top structures, these activities occurred rapidly, possibly on the order of less than a century or two. Thus, it would seem the reoccupation of these sites coincided with dramatic changes in social structure, as well as the ability of individuals or groups to coordinate labor.

Thompson et al. [2] suggest that the cooling and sea-level lowering following AD 500 precipitated a shift in household organization from small family dwellings to large multifamily houses at Pineland. Ethnohistoric documents for Mound Key describe large Calusa houses, such as the one identified archaeologically at Pineland. Specifically, for Mound Key the documents state that in 1697 the total population of the town (ca. 1,000) lived in 16 structures [10, 60]. Thompson et al. [2] speculate that this early shift to larger co-resident family housing was due to the changing distribution of aquatic foods, which required more cooperative labor. They suggest that this labor-pooling set the stage for large-scale landscape manipulation (e.g., canals), as sea levels rose and brought with them more diverse and greater numbers of fish into the estuaries during the Medieval Warm Period (ca. AD 850–1200) [2, 10].

The pattern emerging from our research at Mound Key, Pineland, and other sites suggests a complex, intertwined history of humans and the environment. It appears that changes in household labor organization and the extraction of resources in a fluctuating environment set the stage for larger political shifts and the ability for the Calusa to collectively organize at a much broader scale. That these changes occurred in similar fashion at the two largest Calusa sites in the region suggests that these sites were either in direct competition or that they were integrated early, perhaps in a heterarchical arrangement of early centers [2, 3, 10]. We argue that this reorganization and transformation of the landscape was intimately tied at multiple scales to the production of aquatic surpluses that can only be understood through a long term tracking of the distribution of resources, household structures, site histories, and environmental change.

## Summary and Conclusions

In this paper we take a geoarchaeological approach to investigating the formation of Mound Key, the capital of the Calusa kingdom at the time of European contact in the sixteenth century. We focused on several different questions regarding the nature of formation processes at the site, including the nature of midden-mound formation, overall deposition, and site chronology. To evaluate these issues, we employed coring, excavation, and an extensive radiocarbon dating program. This research underscored the complex nature of dating large-scale sites with extensive shell deposits. In particular, our research at Mound Key illustrates the fact that while certainly dated features provide the most secure contexts, meaning can be gleaned from other so-called less secure contexts, only, however, if close attention is paid to the nature of the context and dates.

Several empirical observations can be generated from our research regarding the nature of settlement at Mound Key. First, it appears that the island was occupied early in its existence, abandoned, and then reoccupied. During Mound Key’s second occupation, its inhabitants substantially altered the landscape by redepositing old midden to form at least the upper portions of the two largest midden-mounds. We argue that this reoccupation and the associated large-scale labor projects are part of a deep history of human-environmental interactions tied to the production of aquatic surpluses.

Thus, our work contributes to the ongoing investigation of the development of political complexity in the region. Based on our research, it would seem that previous interpretive models for the Calusa polity fall short and at best only roughly characterize the nature of its emergence. Simple productivity arguments regarding sea-level and population peaks at best only roughly identify the phenomena at play in the development of these fisher-gatherer-hunter political systems in the region. For example, Widmer [61] suggests that by AD 800 the Calusa region had reached carrying capacity and that political organization had shifted to a system based on inherited status. Most interestingly, it is at this time that we believe that some of the larger sites are in fact abandoned [3]. In addition, general evolutionary aggrandizer models of the development of political complexity [62–64], similarly fail to capture the full range of historical factors important to understand the emergence of these systems, as we outline above. These top-down approaches are fueled by traditional links between social evolution and surplus production and few studies have substantive engagement at the long term history of surplus production, as it relates to changes among fisher-gatherer-hunters as a whole [3].

We argue that the pattern emerging for the Calusa region is by far more historically situated and contingent upon a dynamic interplay among household reorganization, surplus production, and environmental dynamics than previously imagined by scholars. From our perspective, the role of environmental change and surplus production in daily life needs to be given more weight in such studies, as well as the potential for unintended consequences regarding the development of vertical inequalities related to surplus production as well as historically contingent factors related to large scale environmental change. Our research is an ongoing agenda designed to evaluate the nature of surplus production, environmental change, and the development of the powerful Calusa polity at the time of European contact.

## Supporting Information

S1 Table Corrected and modeled probability distributions for all AMS and conventional radiocarbon dates for Mound Key. Marine dates were calibrated using Marine13 with a Delta R of -5 +/-20 and all terrestrial dates were calibrated using IntCal13 curve [65] in a simple phase model in OxCal [66]. All provenience information is provided in the first column and is coded following the site number (8LL2) with operation and unit or core designation (e.g., C-1 for operation C unit 1 or C25 for core 25), followed by level or stratum and feature number and field specimen number (FS) if appropriate.
(XLSX)
